# Abnormal glycogen chain length pattern, not hyperphosphorylation, is critical in Lafora disease

**DOI:** 10.15252/emmm.201707608

**Published:** 2017-05-23

**Authors:** Felix Nitschke, Mitchell A Sullivan, Peixiang Wang, Xiaochu Zhao, Erin E Chown, Ami M Perri, Lori Israelian, Lucia Juana‐López, Paola Bovolenta, Santiago Rodríguez de Córdoba, Martin Steup, Berge A Minassian

**Affiliations:** ^1^ Program in Genetics and Genome BiologyThe Hospital for Sick Children Research InstituteTorontoONCanada; ^2^ Glycation and Diabetes, Mater Research Institute, Translational Research InstituteThe University of QueenslandBrisbaneQldAustralia; ^3^ Institute of Medical ScienceUniversity of TorontoTorontoONCanada; ^4^ Centro de Investigaciones BiológicasConsejo Superior de Investigaciones Científicas and Ciber de Enfermedades RarasMadridSpain; ^5^ Centro de Biología Molecular Severo OchoaCSIC‐UAM and Ciber de Enfermedades RarasUniversidad Autónoma de MadridMadridSpain; ^6^ Division of Neurology, Department of PediatricsUniversity of Texas SouthwesternDallasTXUSA

**Keywords:** glycogen chain length, glycogen phosphorylation, Lafora disease, laforin, malin, Genetics, Gene Therapy & Genetic Disease, Metabolism, Neuroscience

## Abstract

Lafora disease (LD) is a fatal progressive epilepsy essentially caused by loss‐of‐function mutations in the glycogen phosphatase laforin or the ubiquitin E3 ligase malin. Glycogen in LD is hyperphosphorylated and poorly hydrosoluble. It precipitates and accumulates into neurotoxic Lafora bodies (LBs). The leading LD hypothesis that hyperphosphorylation causes the insolubility was recently challenged by the observation that phosphatase‐inactive laforin rescues the laforin‐deficient LD mouse model, apparently through correction of a general autophagy impairment. We were for the first time able to quantify brain glycogen phosphate. We also measured glycogen content and chain lengths, LBs, and autophagy markers in several laforin‐ or malin‐deficient mouse lines expressing phosphatase‐inactive laforin. We find that: (i) in laforin‐deficient mice, phosphatase‐inactive laforin corrects glycogen chain lengths, and not hyperphosphorylation, which leads to correction of glycogen amounts and prevention of LBs; (ii) in malin‐deficient mice, phosphatase‐inactive laforin confers no correction; (iii) general impairment of autophagy is not necessary in LD. We conclude that laforin's principle function is to control glycogen chain lengths, in a malin‐dependent fashion, and that loss of this control underlies LD.

## Introduction

Lafora disease (LD) is a recessively inherited teenage‐onset severe and fatal progressive myoclonus epilepsy. Its etiopathological hallmark is the Lafora body (LB), present throughout the brain in profuse ever‐increasing numbers and sizes (Lafora & Glueck, 1911; Minassian, 2001). LBs are overwhelmingly composed of an abnormally formed glycogen (Sakai *et al*, 1970) and likely result from gradual precipitation, aggregation and accumulation of abnormal glycogen.

Normally glycogen consists of highly and regularly branched roughly sphere‐shaped molecules, each composed of up to 55,000 glucose units. It is mainly synthesized by coordinated actions of glycogen synthase (GS), which elongates glucan chains forming α1‐4 interglucosidic linkages, and glycogen branching enzyme, which detaches a part of each newly formed chain and reattaches it to another glucan chain through an α1‐6 linkage. Glycogen is essentially degraded by glycogen phosphorylase and glycogen debranching enzyme. Glucan chains of glycogen have a wide chain length distribution with an average of ~13 units (Roach *et al*, 2012; Nitschke *et al*, 2013). High frequency of branching points and relatively short chains are critical for glycogen solubility. They prevent neighbouring chains from forming double helices, which otherwise exclude water, reduce the water solubility of the interacting chains and finally lead the entire molecule to precipitate. Incidentally, the main component of plant starch amylopectin is insoluble precisely because it is composed of longer chains that through clustered arrangement of branch points form crystalline areas of double helices (Roach *et al*, 2012; Cenci *et al*, 2014). Glycogen and amylopectin possess phosphate esters bound to a very small number of glucose units, in glycogen's case at carbon positions C2, C3 and C6 (Tagliabracci *et al*, 2011; Nitschke *et al*, 2013). The kinase(s) responsible for glycogen phosphorylation are not known, except that GS itself has been reported to be involved in C3 phosphorylation through what so far has been considered an undesirable side reaction of the enzyme (Tagliabracci *et al*, 2011; Contreras *et al*, 2016).

The abnormal glycogen constituting LBs has three characteristics: (i) its chain length distribution contains longer chains than normal glycogen, (ii) its phosphate levels are elevated, and (iii) its quantity is high, the latter reflecting the profuse deposition as LBs (Tagliabracci *et al*, 2008; Nitschke *et al*, 2013). LD is mainly caused by loss‐of‐function mutations in either the *EPM2A* (laforin) or *EPM2B* (malin) gene, and knockout of either in mouse recapitulates the disease (Minassian *et al*, 1998; Ganesh *et al*, 2002; Chan *et al*, 2003; Turnbull *et al*, 2010). Laforin is a glycogen phosphatase, its absence resulting in progressive glycogen hyperphosphorylation (Worby *et al*, 2006; Tagliabracci *et al*, 2008). Malin is a ubiquitin E3 ligase (Gentry *et al*, 2005), which in cell culture experiments, in a laforin‐dependent fashion, diminishes both GS and a protein involved in GS activation, R5/PTG, through proteasomal degradation (Vilchez *et al*, 2007; Worby *et al*, 2008). Malin, again in a laforin‐dependent manner, also diminishes a second protein that participates in GS activation, R6, in this case through autophagic degradation (Rubio‐Villena *et al*, 2013). Laforin and malin form a functional complex, the former (but not the latter) possessing a carbohydrate binding domain through which the complex is targeted to glycogen (Gentry *et al*, 2005; Lohi *et al*, 2005).

From the above results, two main hypotheses were advanced to explain the abnormal glycogen formation in LD: (i) absence of laforin or malin leads to increased GS activity, which outpacing branching leads to the formation of glycogen with long chains promoting water insolubility (Vilchez *et al*, 2007); and (ii) laforin deficiency results in non‐removal of erratically introduced phosphate and thus hyperphosphorylation of glycogen, which via an unknown mechanism alters its structure promoting glycogen precipitation (Tagliabracci *et al*, 2008; Roach, 2015). Both hypotheses, however, were challenged by more recent findings. (i) Tissues from laforin and malin knockout mice were shown to have unaltered GS activity (Tagliabracci *et al*, 2008; DePaoli‐Roach *et al*, 2010). (ii) When phosphatase‐inactive laforin was overexpressed in laforin knockout mice, LBs no longer formed and the mice were rescued from LD (Gayarre *et al*, 2014). The latter result was particularly provocative. It suggested that laforin's chief and only established physiological function (glycogen dephosphorylation) and the major and distinguishing pathological feature of LD glycogen (progressive hyperphosphorylation) were irrelevant to LB formation and LD.

Gayarre *et al*, however, left a number of critical questions unaddressed before the field could make a decided paradigm shift away from the centrality of glycogen phosphate in LB formation and LD towards exploring alternative disease mechanisms. They did not measure glycogen phosphate. It can, therefore, not be excluded that the overexpressed *in vitro‐*inactive laforin had retained some phosphatase activity *in vivo* or that there was a small amount of wild‐type (WT) laforin somehow still expressed in the transgenic mice. Laforin's phosphatase activity is catalysed by a cysteine residue (C265 in mouse, C266 in human), which Gayarre and colleagues had mutated to serine to inactivate the enzyme. Recently, crystal structures of laforin were determined (Raththagala *et al*, 2015; Sankhala *et al*, 2015) suggesting the presence of a putative second phosphatase site in laforin (C168 in mouse, C169 in human). The C168 site could be active *in vivo*, especially when overexpressed, and explain the rescue by laforin mutated only at C265.

The other two features specific to LD glycogen, chain length distribution and quantity, were also outside the scope of the above study and had not been analysed. As such, conclusions about the mechanism conferring rescue from LBs remained tentative. It was possible, for example, that the rescued mice did accumulate abnormal glycogen, which, coated with a large amount of hydrophilic protein (overexpressed laforin), remained soluble and did not coalesce into pathologically observable LBs.

In this work, we address all these points by characterizing glycogen in both laforin‐ and malin‐deficient mice as affected by overexpressed WT and phosphatase‐inactive laforin. Our results combine with previous studies to allow the proposal of a unifying hypothesis of LB pathogenesis in both laforin‐ and malin‐deficient LD.

## Results

### Amounts and molecular parameters of muscle glycogen in *Epm2a* ^−/−^ mice overexpressing phosphatase‐inactive laforin

**Figure 1:**
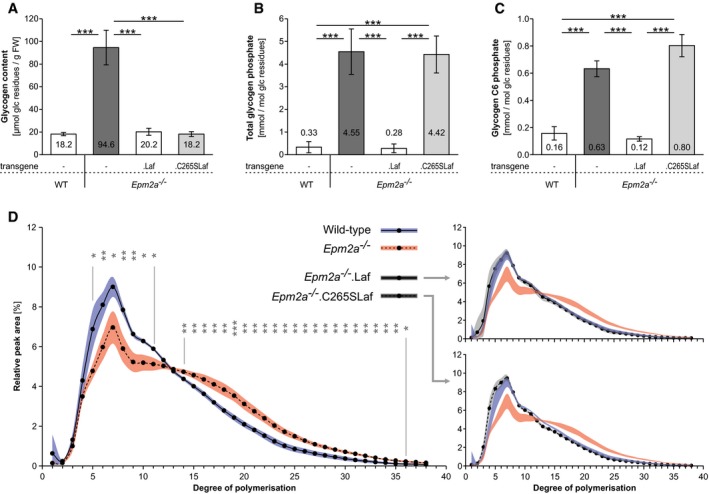
Phosphatase‐inactive laforin does not correct LD muscle glycogen hyperphosphorylation, but does correct its chain length distribution and prevents abnormal glycogen accumulation Laf indicates WT laforin and C265Laf indicates phosphatase‐inactive laforin transgenes, respectively, expressed in indicated LD mouse model (*Epm2a* ^−/−^).
Glycogen content.Glycogen total phosphate content.Glycogen carbon C6 phosphate content.Chain length distribution analyses.Data information: Means of > 5 biological replicates are presented as bars in (A–C) or as circles in (D). Standard deviations are presented as error bars in (A–C), or as blue (WT), red (*Epm2a* ^−/−^) or grey shades (*Epm2a* ^−/−^.Laf and *Epm2a* ^−/−^.C265SLaf) in (D). Asterisks, statistical significance by ANOVA and *post hoc* analyses (*P < 0.05, **P < 0.01, ***P < 0.001; Appendix Table S1).

We quantified total glycogen from skeletal muscle from the actual mice used in the Gayarre *et al* study (Gayarre *et al*, 2014). In *Epm2a* ^−/−^ mice, muscle glycogen content was fivefold increased, as in previous studies (Tagliabracci *et al*, 2008). In *Epm2a* ^−/−^ mice overexpressing WT laforin (*Epm2a* ^−/−^.Laf), muscle glycogen content was normal. In *Epm2a* ^−/−^ mice overexpressing phosphatase‐inactive laforin (*Epm2a* ^−/−^.C265SLaf), muscle glycogen content was also normal (Fig 1A), indicating that the prevention of LBs (which are aggregates of insoluble glycogen) by phosphatase‐inactive laforin (Gayarre *et al*, 2014) is indeed due to the prevention of generating insoluble glycogen, as opposed to merely maintaining the excess abnormal glycogen in a soluble state.

We measured total glycogen phosphate in the above tissues, which in *Epm2a* ^−/−^ mice was 14‐fold increased, as in previous studies (Tagliabracci *et al*, 2008). Levels were normal in *Epm2a* ^−/−^.Laf mice, but in *Epm2a* ^−/−^.C265SLaf mice glycogen phosphate was just as high as in *Epm2a* ^−/−^ mice (Fig 1B). This indicates that phosphatase‐inactive laforin indeed has no phosphatase activity *in vivo* and does not normalize the hyperphosphorylation characteristic of LD glycogen. This in turn indicates that glycogen hyperphosphorylation does not cause the generation of insoluble glycogen.

Measurement of total glycogen phosphate requires relatively large amounts of purified glycogen (see Materials and Methods). Of the three glucosyl carbons phosphorylated in glycogen, C6 phosphorylation is the only one for which a sensitive method has been developed that allows site‐specific quantification. Moreover, C6 phosphate can be measured in very small amounts of glycogen, and as all three phosphorylation sites are proportionally high in LD (DePaoli‐Roach *et al*, 2015; Roach, 2015), it can be used as a measure of total glycogen phosphorylation. While sufficient amounts of glycogen can be purified from skeletal muscle for total glycogen phosphate measurement, this is currently unfeasible for brain glycogen, where C6 phosphorylation only would be quantified as an indicator of the total. Leading up to the brain studies (next sections), we measured C6 glycogen phosphate in skeletal muscle and found that it is, as expected, like total glycogen phosphate, increased in both the *Epm2a* ^−/−^ and *Epm2a* ^−/−^.C265SLaf mice (Fig 1C).

Besides glycogen amount and phosphate content, the third feature that distinguishes LD from WT glycogen is chain length distribution. We measured skeletal muscle glycogen chain lengths in the above genotypes and found that the chain length distribution in *Epm2a* ^−/−^ mice is abnormal with a significantly increased proportion of longer chains, while in *Epm2a* ^−/−^.C265SLaf mice (and of course *Epm2a* ^−/−^.Laf mice) the chain length distribution is normal, indicating that phosphatase‐inactive laforin rescues the glycogen chain length abnormality characteristic of LD glycogen (Fig 1D).

To summarize, phosphatase‐inactive laforin does not correct the hyperphosphorylation of LD muscle glycogen, but does normalize the glycogen's chain length distribution preventing abnormal glycogen accumulation and LB formation.

### Brain glycogen in *Epm2a* ^−/−^ and *Epm2b* ^−/−^ mouse lines overexpressing phosphatase‐inactive laforin

#### Generation of new mouse line

The implications of the Gayarre *et al* (2014) study and our above results are major: Laforin's phosphatase function is dispensable and glycogen hyperphosphorylation is not pathogenic. These results run counter to a leading LD hypothesis, in which glycogen phosphorylation is considered a damaging side product of GS activity that needs to be cleared by laforin (Tagliabracci *et al*, 2011; Raththagala *et al*, 2015; Roach, 2015; Contreras *et al*, 2016; Turnbull *et al*, 2016). To rule out the possibility of an artefact in the Gayarre *et al* mice, we generated a whole new set of *Epm2a* ^−/−^, and this time also *Epm2b* ^−/−^ mice overexpressing phosphatase‐inactive laforin. Phosphatase‐inactive human laforin (C266SLaf) was used, in contrast to the phosphatase‐inactive murine laforin (C265SLaf) used by Gayarre *et al* (2014). We did so by crossing WT mice overexpressing C266SLaf available in our laboratory (Chan *et al*, 2004) with *Epm2a* ^−/−^ (Ganesh *et al*, 2002) and *Epm2b* ^−/−^ (Turnbull *et al*, 2010) mice. The resultant transgenic animals are termed *Epm2a* ^−/−^.C266SLaf and *Epm2b* ^−/−^.C266SLaf, respectively.

**Figure 2:**
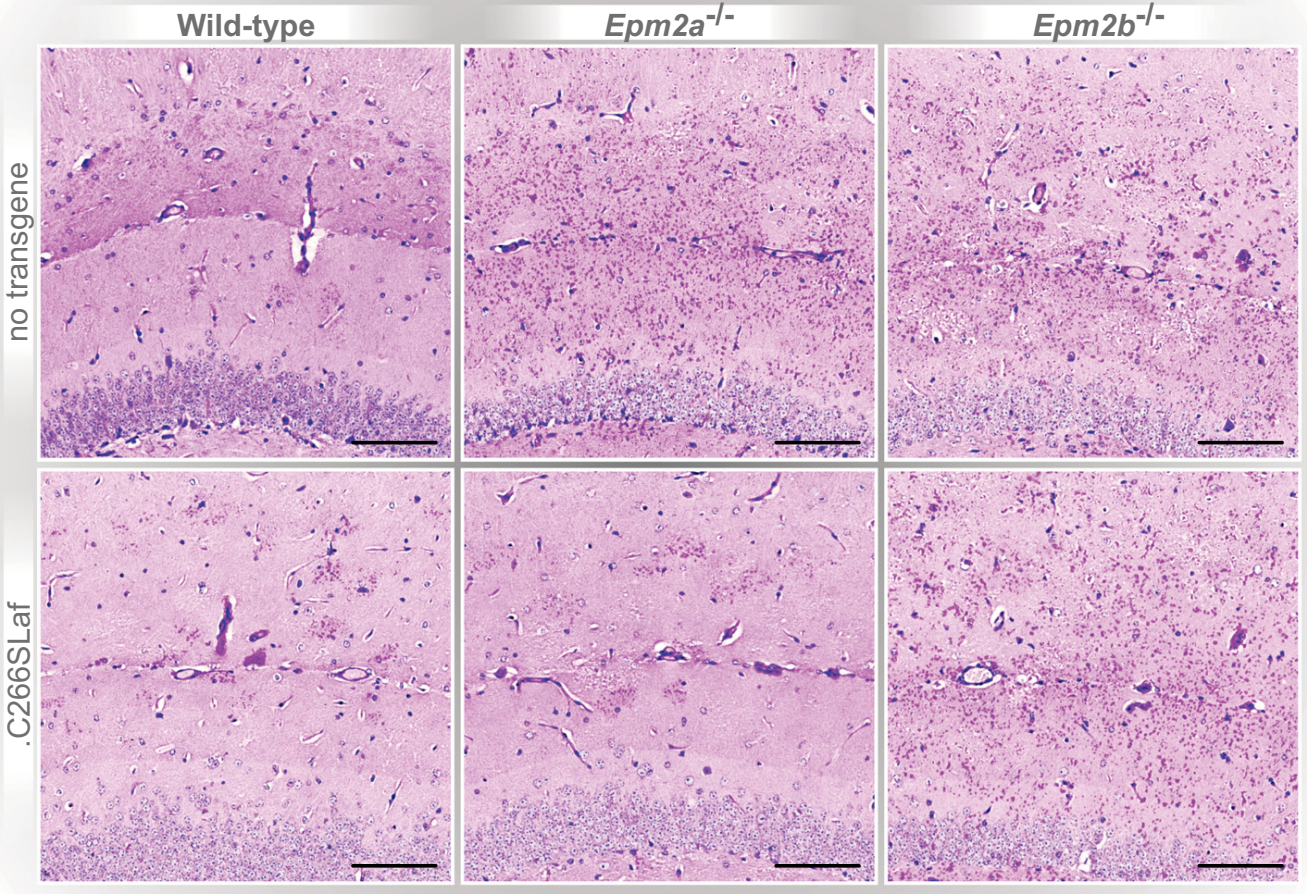
Phosphatase‐inactive laforin prevents *Epm2a* ^−/−^ but not *Epm2b* ^−/−^ from LB formation Representative PASD‐stained sections of hippocampi from WT, *Epm2a* ^−/−^ and *Epm2b* ^−/−^ mice in the absence or presence of the human C266S mutated laforin transgene (.C266SLaf). Scale bar equals 100 μm.Source data are available online for this figure.

We simultaneously analysed glycogen and LBs from this new line and from the Gayarre *et al Epm2a* ^−/−^.C265SLaf mice, focusing on brain, the disease‐relevant organ. Consistency of findings in Gayarre *et al* and our mice would confirm phosphatase‐inactive laforin's effect on glycogen and obviate results arising from any particularities of each mouse line studied. The differences between the mouse lines are transgene species (as mentioned above), presence or absence of a tag on the transgene protein, expression level, and mouse background (Chan *et al*, 2004; Gayarre *et al*, 2014). We had previously generated the C266SLaf mice (Chan *et al*, 2004) in an attempt to create an LD mouse model through a dominant‐negative approach. The phosphatase‐inactive laforin (C266SLaf) was supposed to outcompete WT laforin's phosphatase activity and lead to an effective loss of the endogenous laforin function. In fact, the resultant mice did have a few LBs and we reported this mouse as a mild, only‐pathological, model of LD, with no neurological phenotype (Chan *et al*, 2004). Compared to the laforin (and malin) knockout mice (*Epm2a* ^−/−^ and *Epm2b* ^−/−^ *,* respectively) (Ganesh *et al*, 2002; Turnbull *et al*, 2010), the number of LBs occurring in this model is orders of magnitude lower (Fig 2), and negligible for purposes of the present experiments.

#### Glycogen and LB analyses

**Figure 3:**
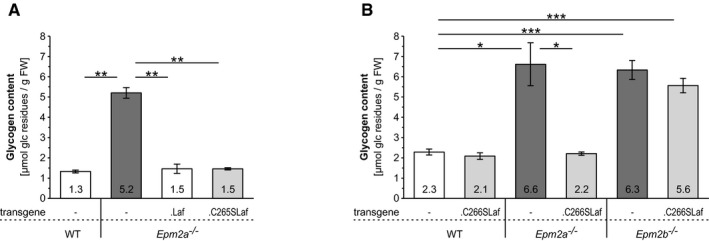
Phosphatase‐inactive laforin rescues *Epm2a* ^−/−^, but not *Epm2b* ^−/−^ abnormal glycogen accumulation in the brain Brain glycogen levels in the presence or absence of WT (.Laf) or C265S mutated (.C265Laf) murine laforin transgene (tissues from the Gayarre *et al*, 2014 study).Brain glycogen levels of WT, *Epm2a* ^−/−^ and *Epm2b* ^−/−^ mice in the presence or absence of human C266S mutated laforin (.C266SLaf) (tissues from the new mice generated in the present study).Data information: Data are presented as means of at least five biological replicates ± SEM. Asterisks, statistical significance by ANOVA and *post hoc* analyses (*P < 0.05, **P < 0.01, ***P < 0.001; Appendix Table S2).

Brain glycogen content was threefold to fourfold increased in both *Epm2a* ^−/−^ mouse lines and in *Epm2b* ^−/−^ mice, as previously published (Tagliabracci *et al*, 2008; DePaoli‐Roach *et al*, 2010; Turnbull *et al*, 2010). Brain glycogen content was normal in *Epm2a* ^−/−^.C265SLaf and *Epm2a* ^−/−^.C266SLaf mice, but remained elevated in *Epm2b* ^−/−^.C266SLaf mice (Fig 3A and B). Thus, overexpressed phosphatase‐inactive laforin corrects the abnormal glycogen accumulation in laforin‐deficient LD but not in malin‐deficient LD. The latter indicates that the rescue in the former is not through some mechanism related to overexpressed protein (somehow physically solubilizing insoluble glycogen) as otherwise the same correction would be expected in the malin‐deficient background.

Consistent with the rescue of the abnormal brain glycogen accumulation in the *Epm2a* ^−/−^.C265SLaf and *Epm2a* ^−/−^.C266SLaf mice, there were no LBs in the brains of these mice. LBs were, however, still present in the *Epm2b* ^−/−^.C266SLaf mice, and to the same extent as in their *Epm2b* ^−/−^ controls (Fig 2), again indicating that phosphatase‐inactive laforin prevents LB formation in laforin‐deficient but not malin‐deficient LD.

**Figure 4:**
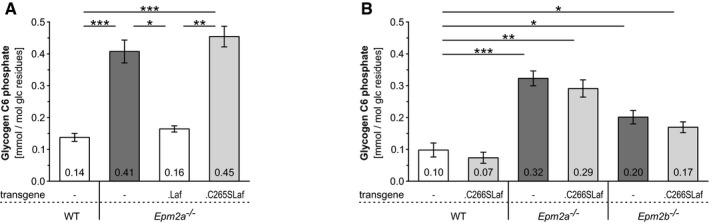
Phosphatase‐inactive laforin does not rescue glycogen hyperphosphorylation in *Epm2a* ^−/−^ or *Epm2b* ^−/−^ brain Brain glycogen carbon C6 phosphate levels in the presence or absence of WT (.Laf) or C265S mutated (.C265Laf) murine laforin transgene.Brain glycogen C6 phosphate levels of WT, *Epm2a* ^−/−^ and *Epm2b* ^−/−^ mice in the presence or absence of human C266S mutated laforin (.C266SLaf).Data information: Data are presented as means of at least five biological replicates ± SEM. Asterisks, statistical significance by ANOVA and *post hoc* analyses (*P < 0.05, **P < 0.01, ***P < 0.001; Appendix Table S3).

We next measured brain glycogen phosphate (via C6 phosphate quantification). Due to particularities of brain tissue, this (and the below glycogen chain length analyses) required extensive new method development, detailed in the Materials and Methods section, and the results represent the first time these analyses have been possible in brain. Brain glycogen phosphate was threefold and twofold elevated in *Epm2a* ^−/−^ and *Epm2b* ^−/−^ mice, respectively, similar to muscle glycogen phosphate (Tagliabracci *et al*, 2008; DePaoli‐Roach *et al*, 2010; Turnbull *et al*, 2010). This increase was not corrected in any of the three phosphatase‐inactive laforin expressing mouse genotypes (*Epm2a* ^−/−^.C265SLaf, *Epm2a* ^−/−^.C266SLaf and *Epm2b* ^−/−^.C266SLaf) (Fig 4). Taken together, the results in this section clearly indicate that hyperphosphorylation of glycogen is not pathogenic nor is it causative of abnormal glycogen accumulation and LB formation.

**Figure 5:**
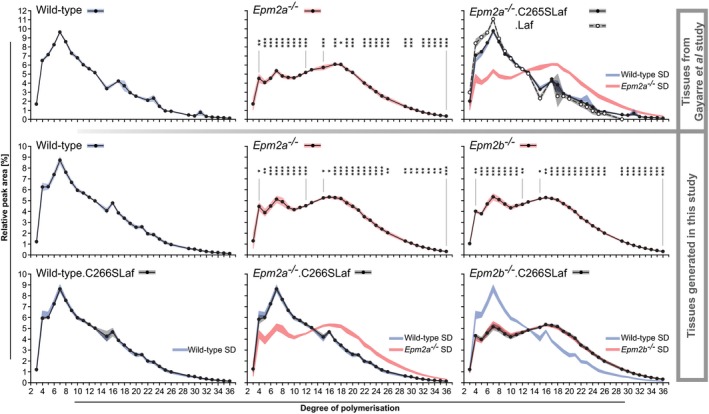
Phosphatase‐inactive laforin rescues brain glycogen chain length distribution in *Epm2a* ^−/−^ but not *Epm2b* ^−/−^ LD Top row from tissues from the Gayarre *et al* (2014) study; bottom two rows from mice generated in the present work. Chain lengths (*x*‐axes) are given as degrees of polymerization (DP), that is, numbers of glucose units per chain. Laf indicates WT laforin transgene expressed in indicated LD genotype. C265SLaf and C266SLaf indicate phosphatase‐inactive murine or human laforin, respectively, expressed in indicated LD genotype. Data are presented as means of relative peak areas for each DP with shades representing standard deviation (SD; blue, WT; red, *Epm2a* ^−/−^ or *Epm2b* ^−/−^; grey, indicated transgenic mice; *n* > 5, for *Epm2a* ^−/−^.Laf samples were pooled). In all panels relating to transgenic mice, in addition to the chain length distribution in these mice, SD shades of WT or respective knockout mutants (*Epm2a* ^−/−^ or *Epm2b* ^−/−^) are shown to allow direct comparisons of the chain length distribution curves. Asterisks, statistical significance by ANOVA and *post hoc* analyses with regard to DP abundances in respective WT glycogen (*P < 0.05, **P < 0.01, ***P < 0.001; Appendix Table S4).

Similar to previous findings in muscle glycogen (Fig 1D; see also Nitschke *et al*, 2013), but even more pronounced, brain glycogen chain length distributions were abnormal, significantly shifted towards longer chains, in *Epm2a* ^−/−^ and *Epm2b* ^−/−^ mice. They were normalized in *Epm2a* ^−/−^.C265SLaf and *Epm2a* ^−/−^.C266SLaf mice (indistinguishable from WT) and remained abnormal in *Epm2b* ^−/−^.C266SLaf (Fig 5). This demonstrates that phosphatase‐inactive laforin can correct the brain glycogen chain length abnormality in laforin‐deficient LD, but not in malin‐deficient LD. These results tease apart, for the first time, the three characteristics of abnormal LD glycogen (glycogen accumulation, hyperphosphorylation, abnormal chain length distribution) and indicate that the altered glycogen chain length distribution, and not hyperphosphorylation, correlates with abnormal glycogen accumulation and LB formation. Moreover, phosphatase‐inactive laforin's ability to rescue the chain length abnormality (and the abnormal glycogen accumulation/LBs) depends on the presence of malin, as this correction does not occur in the malin‐deficient mice (*Epm2b* ^−/−^.C266SLaf). This implies that laforin (irrespective of its phosphatase domain) and malin impact glycogen chain length distribution and prevent LB formation and LD concertedly. It directly links both proteins to glycogen structure *in vivo* and corroborates the close laforin–malin partnership previously suggested by cell culture overexpression experiments (Gentry *et al*, 2005; Lohi *et al*, 2005; Vilchez *et al*, 2007).

#### Effect of phosphatase‐inactive laforin on LD‐associated general defect in autophagy

**Figure 6:**
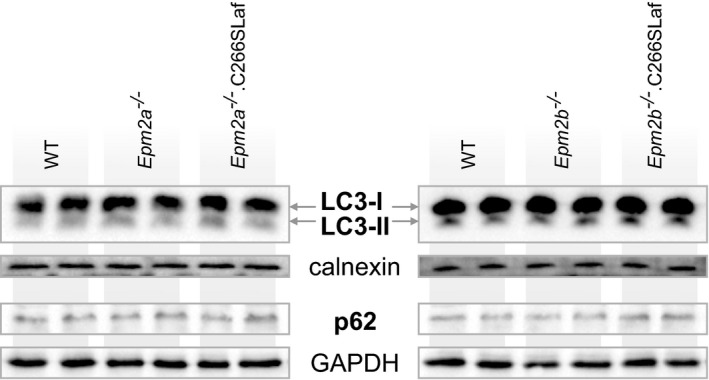
C266S laforin's effect on LD‐associated general defect in autophagy Representative Western blots of autophagy markers LC3 and p62 in protein extracts from laforin‐ and malin‐deficient mouse brains and their respective WT tissues. Loading controls were calnexin for LC3 detection in an autophagosome‐enriched membrane fraction and GAPDH for soluble p62.

LC3 becomes lipidated (i.e. LC3‐I converts to LC3‐II) as it joins proliferating autophagosomes during autophagy, and therefore decreased LC3‐II indicates decreased autophagy. p62 directs ubiquitinated proteins to autophagy, and thus, accumulation of p62 indicates failing autophagy. LD mouse brains were shown to have reduced LC3‐II and increased p62, suggesting that LD mice have impaired autophagy and that this defect underlies LB accumulation (Criado *et al*, 2012). Gayarre and colleagues reported normalization of LC3‐II levels in their *Epm2a* ^−/−^.C265Laf mice, suggesting that phosphatase‐inactive laforin rescues laforin knockout mice by correcting the autophagic defect (Gayarre *et al*, 2014). We tested brain LC3‐II and p62 in the new mice generated for the present study and found no differences in either protein between WT, *Epm2a* ^−/−^, *Epm2b* ^−/−^, *Epm2a* ^−/−^.C266Laf, and *Epm2b* ^−/−^.C266Laf genotypes (Fig 6). These results imply that impaired autophagy may not be a common feature to all LD models and, therefore, it is not a strict requirement for LD.

## Discussion

The report that phosphatase‐inactive laforin rescues laforin‐deficient LD (Gayarre *et al*, 2014) runs contrary to the widely accepted view that the disease is caused by lack of laforin‐mediated clearance of erratic glycogen phosphorylation (Raththagala *et al*, 2015; Roach, 2015; Contreras *et al*, 2016; Turnbull *et al*, 2016). In that publication, however, several important issues had remained unaddressed. First, possibly the rescue could be effected by the mere overabundance of (inactive) laforin solubilizing the malstructured (long‐chained) glycogen. Second, the rescue could have been due to the persistence of residual laforin phosphatase activity, either in the mice considered full laforin knockouts or in the overexpressed laforin transgene. Based on recently published structural information, laforin might possess a heretofore unrecognized second phosphatase site that would have been unaffected by the C265S transgene mutation (Sankhala *et al*, 2015). We now rule all these possibilities out. We show that the same inactive laforin overexpression does not correct the abnormal glycogen accumulation/LBs in malin‐deficient LD and that the correction in laforin‐deficient LD includes normalization of both glycogen chain distribution and content, unequivocally indicating that the rescue has a metabolic, glycogen‐related basis. We also show that the rescue occurs with no reduction in glycogen hyperphosphorylation, ruling out that it is due to any residual laforin phosphatase activity.

**Figure 7:**
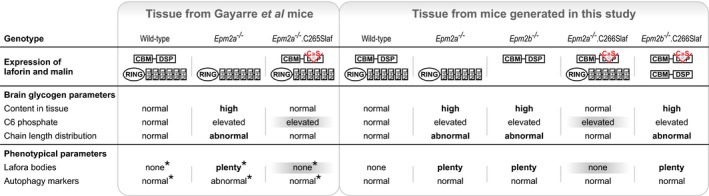
Summary of phosphatase‐inactive laforin's effect in LD mouse model brains LD‐associated phenotypical parameters such as hippocampal LBs and autophagy impairment as well as brain glycogen content are compared with respect to the presence of laforin, malin, and the phosphatase‐inactive laforin transgene. Asterisks mark previously published results (Gayarre *et al*, 2014). Grey shadows emphasize the dissociation between hyperphosphorylation and LB occurrence. Bold letters indicate the association between abnormal chain length distribution and LBs.

Our results show that glycogen hyperphosphorylation does not correlate with LB formation. Hyperphosphorylation is shared by *Epm2a* ^−/−^, *Epm2b* ^−/−^, *Epm2a* ^−/−^.C265SLaf (rescued), *Epm2a* ^−/−^.C266SLaf (rescued), and *Epm2b* ^−/−^.C266SLaf (not rescued) genotypes and can therefore not be the cause of LB formation (Fig 7). The glycogen chain length abnormality, on the other hand, is shared by the *Epm2a* ^−/−^, *Epm2b* ^−/−^, and *Epm2b* ^−/−^.C266SLaf (not rescued) mice, strictly correlating with LB formation and glycogen accumulation (Fig 7). This together with the well‐established facts that longer glucan chains are less water soluble as they form stable double helices and that isolated LBs consist of poorly branched glucans (Sakai *et al*, 1970; Gidley & Bulpin, 1987; Hejazi *et al*, 2009) strongly supports the notion that chain length abnormality is the actual cause of glycogen precipitation and, over time, accumulation into LBs.

Phosphatase‐inactive laforin rescues glycogen precipitation and accumulation in the *Epm2a* ^−/−^.C265SLaf and *Epm2a* ^−/−^.C266SLaf but not *Epm2b* ^−/−^.C266SLaf mice. As the relevant difference between these genotypes is that the latter lacks malin and has an abnormal glycogen chain length pattern, it is safe to conclude that laforin confers its effect strictly together with malin. Previous publications afford insights into the mechanisms through which malin could prevent the generation of glycogen molecules with abnormally long chains. As mentioned, in cell culture experiments malin has consistently been shown to ubiquitinate, in a laforin‐dependent fashion, GS and proteins involved in GS activation (PTG/R5 and R6), targeting them to proteasomal or autophagic degradation (Vilchez *et al*, 2007; Worby *et al*, 2008; Rubio‐Villena *et al*, 2013). On the surface, this mechanism conflicted with the lack of overall elevated GS activity in LD mouse tissues (Tagliabracci *et al*, 2008; DePaoli‐Roach *et al*, 2010). These results can, however, be reconciled if the action of the laforin–malin complex is restricted to a small subset of glycogen molecules. Glycogen is heterogeneous, in both size (Wanson & Drochmans, 1968; Marchand *et al*, 2002; Nitschke *et al*, 2013) and chain length distribution (i.e., some glycogen molecules contain longer chains) (Palmer *et al*, 1983; Nitschke *et al*, 2013). The laforin–malin system would be required only for those molecules/molecule regions in which glucan chains approach thresholds to insolubility. Consistent with this is the observation that laforin's carbohydrate binding domain has greater affinity to longer rather than shorter glucan chains (Chan *et al*, 2004; Lohi *et al*, 2005; Dias *et al*, 2016). A reduced chain elongation would prevent progression towards precipitation, and since the activity would be localized, its absence would not result in detectably increased global GS activity.

Aguado *et al* (2010) reported that their murine *Epm2a* ^−/−^ LD model has an impairment of autophagy, and Criado *et al* (2012) that this impairment presents prior to the appearance of detectable glycogen accumulations/LBs. Gayarre *et al* (2014) showed that phosphatase‐inactive laforin restores autophagy as it rescues LD and proposed that laforin–malin prevents LB formation via autophagy. In contrast and in line with recent publications (Irimia *et al*, 2015; Wang *et al*, 2016), we do not detect presence of an autophagic impairment in the *Epm2a* ^−/−^ and *Epm2b* ^−/−^ animals used to prepare the new transgenic mice generated here and can, therefore, not confirm autophagy correction by laforin–malin. Additionally, other recent work showed that disturbances of autophagy markers present in LD are correctable by genetic reduction of GS activity (Duran *et al*, 2014), suggesting that the autophagy impairment may be secondary to the glycogenosis. Notwithstanding, it is entirely possible that laforin–malin acts locally to eliminate, through autophagy, individual glycogen molecules that are “at risk” of precipitation. In fact, malin was recently shown to be able to mediate K63‐linked ubiquitination, which can result in p62‐mediated substrate targeting to autophagy (Sanchez‐Martin *et al*, 2015). Since laforin preferentially binds glycogen molecules with longer chains (see above), it is conceivable that malin could ubiquitinate laforin through K63 ubiquitination and target laforin‐bound “at‐risk” glycogen molecules to autophagy.

As our results show that phosphorylation is not causative of glycogen precipitation and accumulation, what, if any, are the functions of glycogen phosphates? In starch metabolism, one established function of monophosphate esters of amylopectin is, through their high charges and hydrophilia, to separate amylopectin chains from each other, rendering them fully hydrated, and thus accessible to degrading enzymes (Edner *et al*, 2007; Hejazi *et al*, 2008; Kötting *et al*, 2009; Zeeman *et al*, 2010). As glycogen and amylopectin have a shared chemical basis (both consist of branched alpha‐glucan chains), effects of covalent modifications, such as phosphorylation, are expected to be similar. Phosphorylation should therefore enhance glycogen solubility and may represent a third mechanism to protect against precipitation of long‐chained “at‐risk” molecules. It is well established that neither starch‐degrading exo‐amylases nor glycogen phosphorylase can proceed beyond a phosphorylated glucosyl residue (Lomako *et al*, 1994; Kötting *et al*, 2009; Cenci *et al*, 2014; Emanuelle *et al*, 2016). Laforin's phosphatase role may be, similar to starch phosphatases (Gentry *et al*, 2007; Kötting *et al*, 2009), to remove the phosphates to allow reshortening of chains in “at‐risk” molecules and during glycogen digestion. Consistent with this is the recent demonstration that glycogen dephosphorylation by laforin occurs principally during glycogen degradation (Irimia *et al*, 2015). In the absence of laforin's phosphatase activity, cycles of partial glycogen digestion and resynthesis would lead to the time‐dependent phosphate accumulation that characterizes LD glycogen.

In summary, our results combine with recent work (Gayarre *et al*, 2014) to suggest that laforin's principle LD relevant function is mediated through malin and directed to preventing glycogen molecules with hyperextended chains. In the absence of either protein, some glycogen molecules at a time precipitate and gradually over time aggregate and amass into LBs, which, reaching a certain threshold profusion (at ~14 years of age in humans), initiate, and then drive the progressive myoclonus epilepsy.

## Materials and Methods

### Mouse lines, tissue collection, and histological stain for LBs

Tissues from *Epm2a* ^−/−^.C265SLaf mice and their controls (see Results) were the same as utilized in Gayarre *et al* (2014; C57BL/6J). These mice were 9–12 months old and of both sexes. *Epm2a* ^−/−^.C266SLaf and *Epm2b* ^−/−^.C266SLaf mice and controls (see Results) were obtained following the crossing of both *Epm2a* ^−/−^ (Ganesh *et al*, 2002; C57BL/6J) and *Epm2b* ^−/−^ (Turnbull *et al*, 2010; C57BL/6J) mice with the C266SLaf mice described previously (Chan *et al*, 2004; 129SvJ). Mice were housed with environmental enrichment in ventilated cages at 20–22°C, fed a high‐quality, commercially available diet, water being automatically delivered. Brains of male and female mice were harvested after cervical dislocation of at least six 9‐ to 12‐month‐old mice from each genotype, split along the sagittal plane, one hemisphere immediately frozen in liquid nitrogen, the other fixed in 10% [w/v] formalin. The latter was embedded in paraffin, sectioned and stained using the periodic acid–Schiff diastase method (PASD; Chan *et al*, 2004). All animal procedures were approved by The Centre for Phenogenomics Animal Care Committee and are in compliance with the CCAC Guidelines and the OMAFRA Animals for Research Act.

### Glycogen determination

Glycogen content was determined essentially as described previously (Suzuki *et al*, 2001). Briefly, frozen tissue was ground in liquid nitrogen, boiled in 30% [w/v] KOH, and precipitated thrice in 67% [v/v] ethanol and 15 mM LiCl (at least 1 h at −30°C). After redissolving in water, aliquots were digested with amyloglucosidase (Megazyme) in 80 mM sodium acetate buffer pH 4.5. Liberated glucose was determined enzymatically (Lowry & Passonneau, 1972).

### Glycogen purification from muscle for phosphate determination

Quantification of total glycogen‐bound phosphate requires high glycogen purity, because its relative amounts are very low and non‐glycogen derived phosphoesters, for example, in nucleic acids, can severely affect the determination. Extracted muscle glycogen was thus further purified as previously published (Tagliabracci *et al*, 2007). Subsequently, glycogen was repeatedly precipitated in 67% ethanol and 15 mM LiCl (at least 1 h at −30°C) until the absence of nucleic acids could be confirmed by UV–vis spectra of glycogen solutions at concentrations of ~100 mM glucose equivalents.

### Total glycogen phosphate quantification in muscle glycogen

Total glycogen‐bound phosphate was quantified as orthophosphate following enzymatic hydrolysis of all phospho‐monoesters. To facilitate complete orthophosphate release, highly purified muscle glycogen was incubated with phosphatase together with two glycogen hydrolysing enzymes. In a final volume of 65 μl, the reaction mixture contained 100 or 200 μg glycogen, 2.5 U Antarctic phosphatase (NEB), 0.1 U α‐amylase (Roche), 2 U amyloglucosidase (Megazyme), 50 mM bis–Tris–propane pH 6.0, 1 mM MgCl_2_, and 0.1 mM ZnCl_2_. Following overnight incubation at 37°C, 40 μl of this reaction mixture, and orthophosphate standards for calibration, was used to quantify ortho‐phosphate enzymatically as described previously (Nelson & Kaufman, 1987). Following the addition of equal volume of a buffer pH 7 (100 mM imidazole–HCl, 10 mM MgCl_2_, 5 mM EDTA), in a total volume of 80 μl, orthophosphate, via G1P, is converted to G6P using 0.02 mg rabbit phosphorylase a (Sigma), 67 μg purified rabbit muscle glycogen, and 1 U phosphoglucomutase (Sigma). Fluorescence (ex340/em470) was read until stable, first, after the addition of 150 μl 20 mM Tris pH 8, 60 μM NADP, and again, after the addition of 0.4 U G6PDH (Roche) in 5 μl 20 mM Tris pH 8. The G6PDH‐dependent increase in fluorescence was used to calculate the orthophosphate amounts in samples originally containing 100 or 200 μg glycogen. Glucose‐based phosphate amounts of the latter two measurements for each sample were averaged and deviated < 5% indicating that the enzymatic orthophosphate release was complete.

### Glycogen purification from brain

Initial attempts to quantify C6 phosphate in brain glycogen were unsuccessful when following an isolation procedure similar to that used for muscle glycogen. Unexpected high values of apparent glucosyl C6 phosphate residues in hydrolysates of brain glycogen indicated the presence of a contaminant that greatly affected the measurements. The ratio of contaminant to glycogen was much higher in samples with low glycogen content, such as WT, suggesting that the contaminant is unrelated to glycogen but derives from other constituents of brain (whose glycogen content appears to be low as compared to other organs). Further experiments confirmed that the contaminant is very likely glucose 6‐phosphate released during acid hydrolysis from a glycan‐containing compound other than glycogen. This contamination is relevant only for tissues with low glycogen content, such as brain. Systematic experiments were conducted to remove the contaminant while retaining the little glycogen using spare WT mouse brains. After the extraction of glycogen from 100 to 400 mg ground frozen brain tissue in 30% KOH and three consecutive precipitations in 67% ethanol and 15 mM LiCl (see extraction of muscle glycogen for glycogen determination), the following procedure was established for all brain glycogen samples analysed in this study. The resulting pellet, containing glycogen, most likely proteins, and the contaminant, was resuspended in 100 μl water and subsequently extracted with 900 μl 2:1 methanol:chloroform [v/v] by vigorous mixing, incubation for 25 min at room temperature (RT) on a rotator, and 10 min at 85°C. After cooling to RT, the suspension was centrifuged for 20 min at 20°C and 16,000 × *g*, the supernatant discarded and the pellet dried briefly at 85°C. The extraction procedure was repeated twice, followed by resuspending the dry pellet in 300 μl water. After 20‐min incubation at 95°C with intermittent agitation, the solubilized glycogen was recovered in the supernatant (S1) obtained by centrifugation at RT (16,000 × *g* for 20 min). The pellet was extracted with 100 μl 0.5 M KOH at 95°C for 45 min with intermittent agitation. Subsequently, the extract, cooled to RT, was centrifuged (20 min, RT, 16,000 × *g*), and the resultant supernatant (S2) was combined with the solubilized glycogen (S1). The combined supernatants (S1 + S2) were subjected to several rounds of repeated ethanol precipitation (1,400 μl 67% ethanol, 15 mM LiCl, 70 min at −30°C), 20 min centrifugation at 4°C and 16,000 × *g*, carefully discarding the supernatant, and resolubilization of the pellet in water followed by 10‐min heat treatment (95°C, intermittent agitation). The ethanolic supernatant harbours the contaminant, the amount of which tends to be smaller with each round of precipitation. Glycogen pellets were considered to be contaminant free when the apparent G6P amount in the last ethanolic supernatant was below the detection limit. Using the enzymatic cycling method, the contaminant (apparent G6P) was quantified after drying the ethanolic supernatants (SpeedVac), dissolving the dry contaminant pellets in water, and subsequent acid hydrolysis (see below). Glycogen concentration was determined after dissolving the purified glycogen pellet as described above. As the number of sequential precipitations was adjusted to the persistence of the contaminant in the ethanolic supernatant, glycogen recovery varied but in most cases was between 50 and 80%.

### Glycogen carbon C6 phosphate determination

Aliquots of purified muscle or brain glycogen preparations (see above) were subjected to acid hydrolysis (3 h, 95°C, 0.7 M HCl) and subsequently neutralized using 5 M KOH. G6P was determined alongside G6P standards using the enzymatic cycling assay previously described (Nitschke *et al*, 2013). G6P amounts were based on glucose amounts that were liberated during acid hydrolysis and determined enzymatically (Lowry & Passonneau, 1972).

### Determination of glycogen chain length distributions

Purified muscle or brain glycogen (10–20 μg dry weight) was completely debranched by overnight incubation with 200 U isoamylase (Sigma) in 110 μl 10 mM sodium acetate at pH 5 and 37°C. Following heat inactivation (10 min at 95°C) and centrifugation (10 min at 20,000 × *g*), 90 μl of the supernatant was applied to high‐performance anion exchange chromatography with pulsed amperometric detection (HPAEC‐PAD; ThermoFisher, ICS5000). Separation of the oligoglucan chains was achieved on a CarboPac PA100 column and guard combination (ThermoFisher) at a constant flow rate of 1 ml/min with an elution profile of the two eluents A (150 mM NaOH) and B (150 mM NaOH, 500 mM sodium acetate) as follows: injection after 15 min 95% [v/v] A, 5% [v/v] B equilibration; 5 min 95% A, 5% B; 9 min linear gradient to 70% A, 30% B; 9 min linear gradient to 55% A, 45% B; 37 min linear gradient to 33% A, 67% B; 10 min 100% B. Chromatograms were analysed with Dionex Chromeleon software (version 7.2). For each chain length (degree of polymerization), relative peak areas were determined, which were then averaged among at least five biological replicates allowing the calculation of standard deviations (SD). Note that purified glycogen from all available brain samples of the genotype *Epm2a* ^−/−^.Laf was pooled to obtain sufficiently large amounts of glycogen for the chain length distribution analysis. SD at each chain length as a measure of biological variance for this genotype was estimated by multiplying the average SD of the other genotypes (WT, *Epm2a* ^−/−^, *Epm2a* ^−/−^.C265SLaf) by two. As variances in relative peak areas between those genotypes are mostly equal, the estimated biological variance should include 95% of samples of the *Epm2a* ^−/−^.Laf genotype.

### Analysis of autophagy markers

For the detection of LC3 in brain tissue, a membrane fraction enriched in autophagosomes was prepared as described previously (Chu *et al*, 2009), and for p62 after extracting soluble protein as in Wang *et al* (2007); 15 μg total protein was separated on 14% SDS–PAGE using a Laemmli buffer system. Proteins of interest were detected after transfer to PVDF membranes using primary antibodies NB100‐2220 (LC3, Novus Biologicals), P0067 (p62, Sigma), sc‐25778, and sc‐11397 (GAPDH and calnexin, respectively, Santa Cruz), all at a 1:1,000 dilution.

### Statistical analyses

Except where stated, all biochemical analyses were conducted in at least five biological replicates. Standard deviations (SD) or standard error of mean (SEM) was calculated as indicated. Averages of multiple groups of biological replicates were compared using one‐way analysis of variance (ANOVA) followed by *post hoc* analysis using homoscedastic Student's *t*‐ or heteroscedastic Welch's tests, both unpaired, two‐tailed, and with Holm–Bonferroni correction. The adequate *post hoc* test was selected based on equal or unequal variances of groups tested with *F*‐tests. Significance levels are indicated by asterisks (*P < 0.05, **P < 0.01, ***P < 0.001). For glycogen chain length distribution analyses, significance between genotypes was tested for each individual degree of polymerization.

## Author contributions

BAM, FN, and MS conceived the study in collaboration with SRC. Experiments were conducted by FN, PW, and XZ with help from AMP, LI, LJL, and PB. Data were analysed by FN, EEC, and PW. The manuscript was written by FN, BAM, and MS with critical review from MAS, EEC, and SRC.

## Conflict of interest

The authors declare that they have no conflict of interest.

**The paper explained**

**Problem**

The fatal childhood‐onset epilepsy Lafora disease results from neuronal carbohydrate accumulations (Lafora bodies). Recent partly antagonistic hypotheses suggest that these accumulations originate from glycogen hyperphosphorylation, occurring when the glycogen phosphatase laforin is insufficiently functioning, or through deficiency in general autophagy.

**Results**

Through methodological advances, we are now able to measure brain glycogen structural parameters and show that glycogen chain lengths, not hyperphosphorylation nor deficiency in general autophagy, are critical for the formation of Lafora bodies. The Lafora disease proteins laforin and malin cooperate to regulate glycogen chain length and prevent glycogen malformation and precipitation into neurotoxic Lafora bodies.

**Impact**

The findings in this paper provide further insight into basic principles of glycogen metabolism, especially regarding the role of glycogen phosphate and its chain length distribution. Moreover, current hypotheses on the pathogenesis of Lafora disease are corrected while a view is presented that integrates previously contradicting results, sharpening the understanding of this horrible disease.

## Supporting information



Appendix

Review Process File

Source Data for Figure 2
