# Supplementary material for: Abnormal glycogen chain length pattern, not hyperphosphorylation, is critical in Lafora disease
Source: EMBO Mol Med. 2017 May 23;9(7):906–17. doi: 10.15252/emmm.201707608 (PMC5494504; doi:10.15252/emmm.201707608)
Supplement: Supplementary file 1 — Appendix [file EMMM-9-906-s001.pdf]

Appendix

Manuscript title:

Abnormal glycogen chain length pattern, not hyperphosphorylation, is critical in Lafora disease

Table of contents

**Item**	**Reference in main text**	**Page in this document**
Appendix Table S1	Figure 1	2
Appendix Table S2	Figure 3	3
Appendix Table S3	Figure 4	3
Appendix Table S4	Figure 5	4

Appendix Table S1.

Holm-Bonferroni corrected p values of post-hoc tests in Figure 1. Not all p values are depicted in the figure.

	group 1	group 2	post-hoc p-value
**Figure 1a**	WT	*Epm2a*$^{-/-}$	2.31E-04
	Epm2a$^{-/-}$	*Epm2a*$^{-/-}$.Laf	3.29E-04
	Epm2a$^{-/-}$	*Epm2a*$^{-/-}$.C265SLaf	2.82E-04
**Figure 1b**	WT	*Epm2a*$^{-/-}$	4.96E-05
	WT	*Epm2a*$^{-/-}$.C265SLaf	2.55E-05
	Epm2a$^{-/-}$	*Epm2a*$^{-/-}$.Laf	5.63E-05
	Epm2a$^{-/-}$.Laf	*Epm2a*$^{-/-}$.C265SLaf	2.84E-05
**Figure 1c**	WT	*Epm2a*$^{-/-}$	6.77E-07
	WT	*Epm2a*$^{-/-}$.C265SLaf	8.12E-08
	Epm2a$^{-/-}$	*Epm2a*$^{-/-}$.Laf	4.83E-05
	Epm2a$^{-/-}$.Laf	*Epm2a*$^{-/-}$.C265SLaf	1.08E-05

	degree of polymerisation	post-hoc p-value<br>WT vs. *Epm2a*$^{-/-}$	*Epm2a*$^{-/-}$ vs. *Epm2a*$^{-/-}$.Laf	*Epm2a*$^{-/-}$ vs. *Epm2a*$^{-/-}$.C265SLaf
**Figure 1d**	4	1.90E-01	2.31E-02	3.95E-05
	5	1.22E-02	4.14E-04	3.63E-07
	6	7.05E-03	1.33E-04	1.96E-06
	7	1.30E-02	2.32E-04	7.24E-03
	8	5.05E-03	9.43E-05	7.32E-03
	9	4.47E-03	1.82E-04	1.26E-02
	10	1.44E-02	2.42E-03	2.41E-02
	11	1.95E-02	2.09E-02	7.95E-02
	12	6.09E-02	7.75E-01	4.24E-01
	13	1.30E-01	1.50E-01	1.36E-04
	14	6.58E-03	1.25E-02	2.95E-06
	15	2.69E-03	2.41E-03	9.41E-07
	16	4.96E-03	3.89E-04	2.11E-06
	17	2.59E-03	1.06E-04	1.19E-06
	18	2.31E-03	5.18E-05	2.12E-03
	19	8.75E-04	1.90E-05	6.41E-07
	20	1.34E-03	2.08E-05	1.02E-06
	21	1.31E-03	1.27E-05	5.26E-07
	22	1.19E-03	1.24E-05	4.94E-07
	23	1.75E-03	2.95E-05	3.25E-07
	24	1.47E-03	8.01E-05	3.57E-07
	25	1.39E-03	7.29E-05	2.57E-07
	26	1.42E-03	4.06E-05	1.99E-07
	27	2.00E-03	5.12E-05	2.97E-07
	28	2.43E-03	2.92E-05	1.38E-07
	29	1.94E-03	2.13E-05	2.83E-07
	30	3.42E-03	2.69E-05	4.19E-07
	31	1.57E-03	1.32E-05	5.89E-08
	32	2.88E-03	2.73E-05	1.10E-07
	33	2.00E-03	4.27E-05	4.35E-08
	34	4.05E-03	2.57E-05	4.38E-07
	35	6.46E-03	2.40E-04	4.71E-03
	36	2.27E-02	8.14E-05	9.38E-03
	37	8.46E-02	3.99E-02	2.49E-02

Appendix Table S2.

Holm-Bonferroni corrected p values of post-hoc tests in Figure 3.

	group 1	group 2	post-hoc p-value
**Figure 3a**	WT	*Epm2a*$^{-/-}$	1.58E-03
	Epm2a$^{-/-}$	*Epm2a*$^{-/-}$.Laf	1.37E-03
	Epm2a$^{-/-}$	*Epm2a*$^{-/-}$.C265SLaf	1.47E-03
**Figure 3b**	WT	*Epm2a*$^{-/-}$	1.82E-02
	WT	*Epm2b*$^{-/-}$	1.11E-04
	WT	*Epm2b*$^{-/-}$.C266SLaf	1.21E-05
	Epm2a$^{-/-}$	*Epm2a*$^{-/-}$.C266SLaf	2.60E-02

Appendix Table S3.

Holm-Bonferroni corrected p values of post-hoc tests in Figure 4.

	group 1	group 2	post-hoc p-value
**Figure 4a**	WT	*Epm2a*$^{-/-}$	3.09E-04
	WT	*Epm2a*$^{-/-}$.C265SLaf	6.84E-05
	Epm2a$^{-/-}$	*Epm2a*$^{-/-}$.Laf	1.00E-02
	Epm2a$^{-/-}$.Laf	*Epm2a*$^{-/-}$.C265SLaf	6.31E-03
**Figure 4b**	WT	*Epm2a*$^{-/-}$	1.45E-04
	WT	*Epm2b*$^{-/-}$	1.20E-02
	WT	*Epm2a*$^{-/-}$.C266SLaf	1.63E-03
	WT	*Epm2b*$^{-/-}$.C266SLaf	3.47E-02

Appendix Table S4.

Holm-Bonferroni corrected p values of post-hoc tests in Figure 5. DP is degree of polymerisation. Not all p values are depicted in the figure.

	**Gayarre et al. tissue**		**Tissue generated in this study**			
DP	WT vs. *Epm2a*$^{-/-}$	*Epm2a*$^{-/-}$ vs. *Epm2a*$^{-/-}$.C265SLaf	WT vs. *Epm2a*$^{-/-}$	WT vs. *Epm2b*$^{-/-}$	WT vs. *Epm2b*$^{-/-}$.C266SLaf	*Epm2a*$^{-/-}$ vs. *Epm2a*$^{-/-}$.C266SLaf
3	9.90E-01	1.11E+00	5.10E+00	7.81E-01	1.91E+00	2.85E+00
4	1.02E-03	1.35E-03	4.29E-02	9.14E-03	1.20E-02	2.22E-01
5	1.88E-06	1.09E-05	1.47E-03	2.92E-06	3.73E-04	7.25E-03
6	3.43E-07	3.06E-06	2.79E-04	6.20E-05	4.56E-04	6.17E-04
7	4.26E-08	9.62E-07	1.33E-04	4.96E-05	1.28E-04	1.77E-04
8	2.14E-07	4.34E-06	6.13E-04	9.32E-05	1.97E-04	8.40E-03
9	1.94E-06	1.32E-05	1.04E-04	1.10E-04	2.33E-04	2.04E-04
10	1.69E-06	9.02E-06	8.92E-05	2.20E-04	3.16E-04	8.71E-05
11	1.08E-05	3.86E-05	4.80E-05	3.53E-04	4.56E-04	9.92E-05
12	7.91E-04	2.75E-03	5.19E-05	2.89E-03	3.62E-03	2.02E-04
13	1.34E-03	1.65E-02	4.01E-01	1.17E+00	6.26E-01	3.53E-01
15	3.32E-05	1.05E-04	2.81E-02	3.88E-02	4.67E-02	1.82E-02
16	not applicable	not applicable	2.38E-02	3.71E-03	7.42E-03	2.32E-02
17	2.93E-07	3.74E-06	1.82E-04	8.55E-06	6.21E-05	5.83E-04
18	4.54E-02	4.45E-01	9.24E-05	1.60E-05	8.59E-05	1.52E-04
19	7.04E-07	6.19E-06	3.47E-04	1.74E-05	1.12E-04	1.44E-03
20	8.59E-08	1.46E-06	2.25E-04	2.19E-05	1.50E-04	5.79E-04
21	not applicable	not applicable	9.51E-05	1.35E-04	1.25E-04	1.55E-04
22	3.25E-05	8.19E-05	4.23E-04	2.53E-05	2.19E-04	1.75E-03
23	8.06E-04	4.80E-05	5.31E-04	1.22E-04	6.40E-04	6.49E-04
24	5.74E-05	3.46E-04	8.12E-04	2.57E-05	3.39E-04	2.59E-03
25	1.12E-04	2.07E-04	6.86E-04	2.59E-05	4.45E-04	2.01E-03
26	6.97E-05	3.52E-04	1.41E-03	3.09E-05	6.69E-04	4.94E-03
29	6.32E-05	1.85E-04	1.73E-03	4.32E-05	1.62E-03	6.73E-03
30	2.01E-04	5.93E-04	1.44E-03	4.49E-05	2.03E-03	1.56E-02
31	7.01E-01	2.67E-02	3.84E-03	3.53E-05	3.62E-03	9.69E-03
32	7.40E-04	1.30E-03	1.79E-03	3.81E-05	3.73E-03	6.29E-03
33	3.37E-04	1.07E-03	1.38E-03	3.73E-05	4.93E-03	4.51E-03
34	2.50E-04	9.21E-04	1.63E-03	8.06E-05	9.32E-03	4.53E-03
35	3.33E-05	2.54E-04	1.61E-03	1.26E-04	1.39E-02	8.82E-03
36	5.85E-04	1.51E-03	6.48E-04	2.42E-04	1.60E-02	2.59E-03
