# Supplementary material for: Abnormal glycogen chain length pattern, not hyperphosphorylation, is critical in Lafora disease
Source: EMBO Mol Med. 2017 May 23;9(7):906–17. doi: 10.15252/emmm.201707608 (PMC5494504; doi:10.15252/emmm.201707608)

Figure 2

Wild-type, no transgene

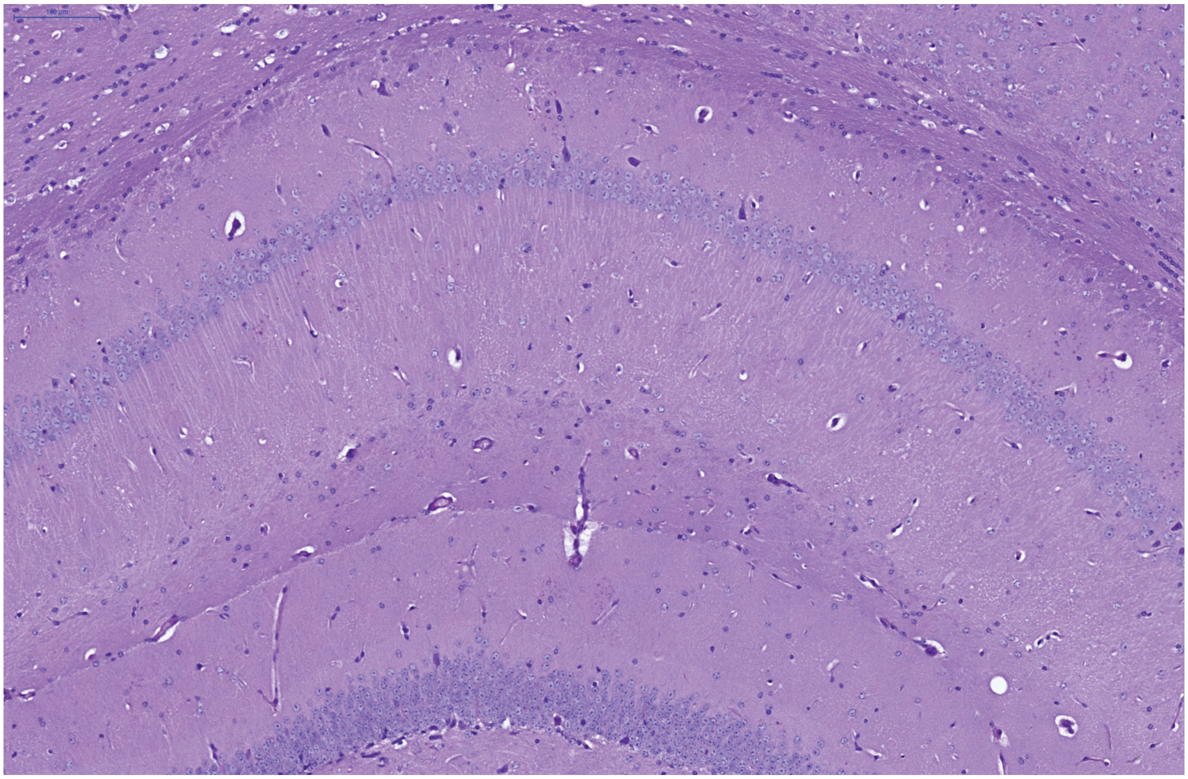

Epm2a$^{-/-}$, no transgene

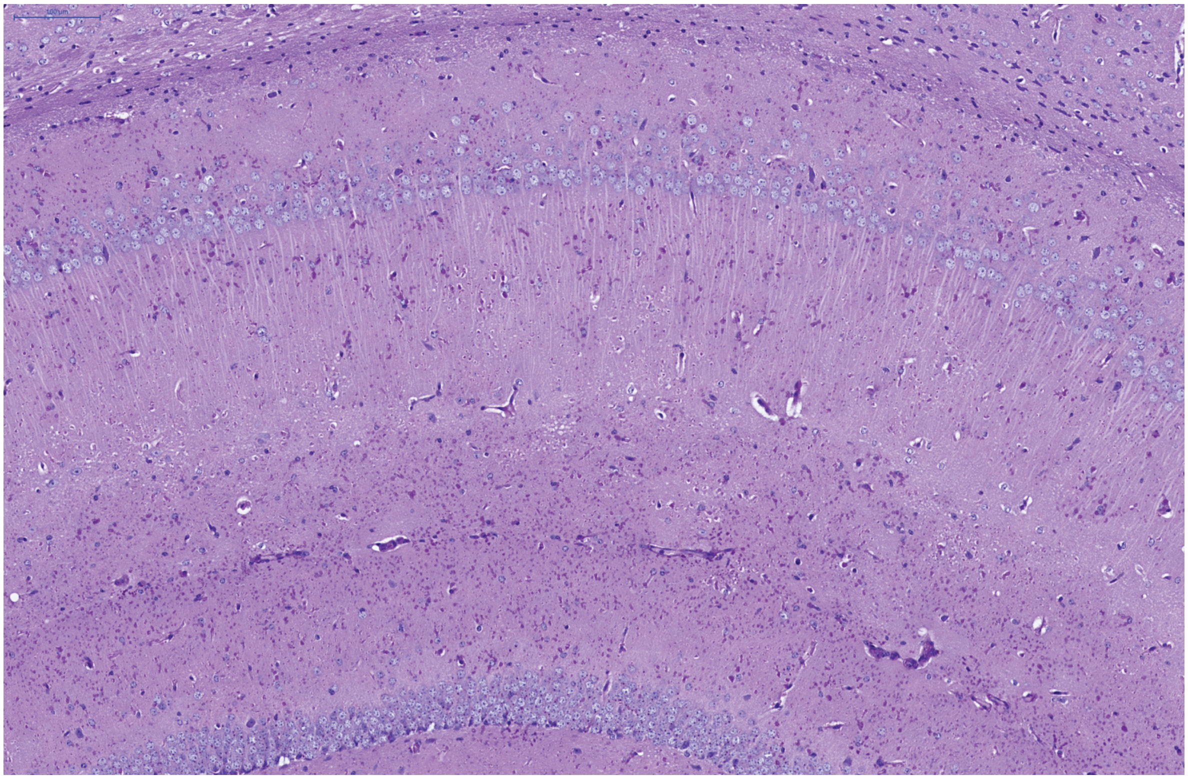

Figure 2

Epm2b$^{-/-}$, no transgene

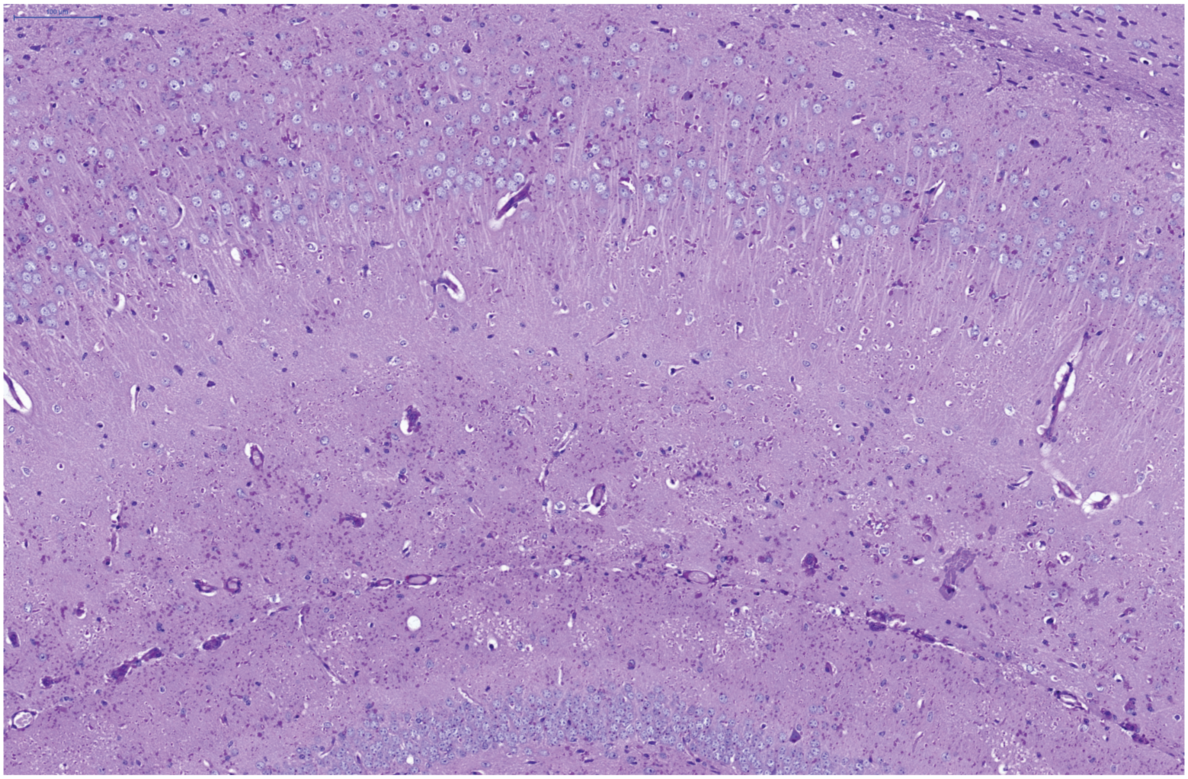

Wild-type.C266SLaf

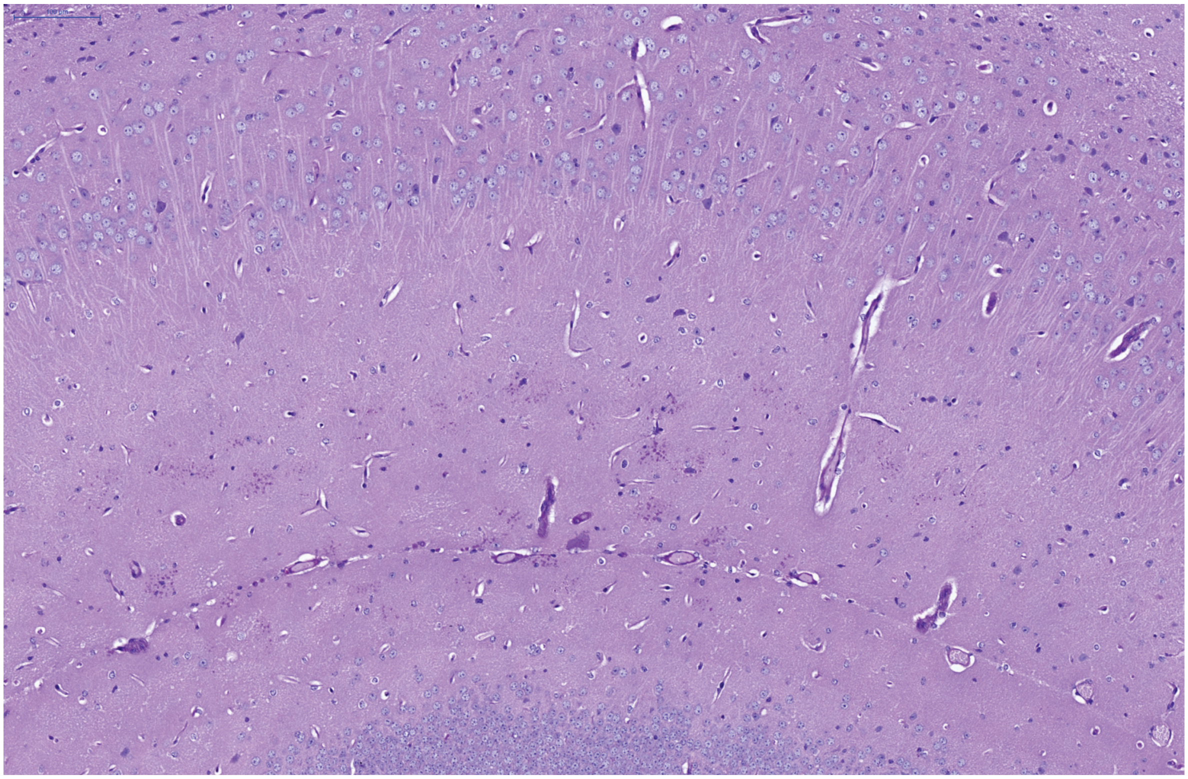

Figure 2

Epm2a$^{-/-}$.C266SLaf

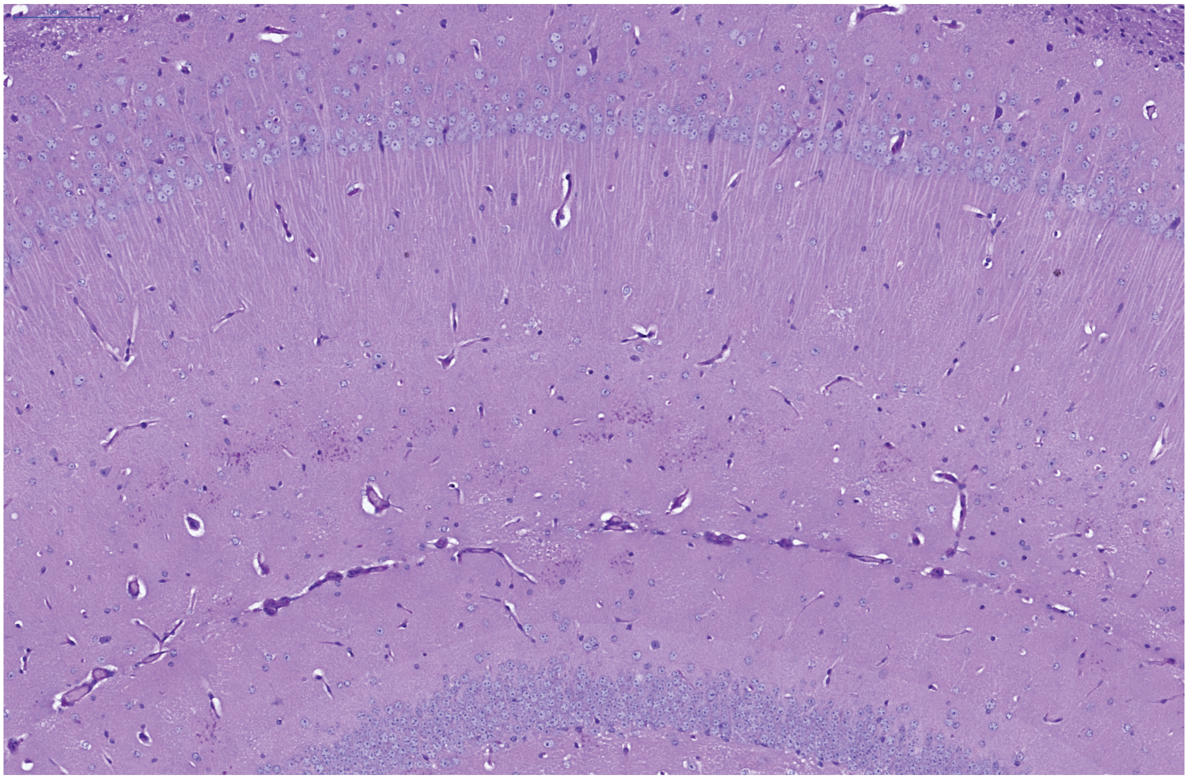

Epm2b$^{-/-}$.C266SLaf

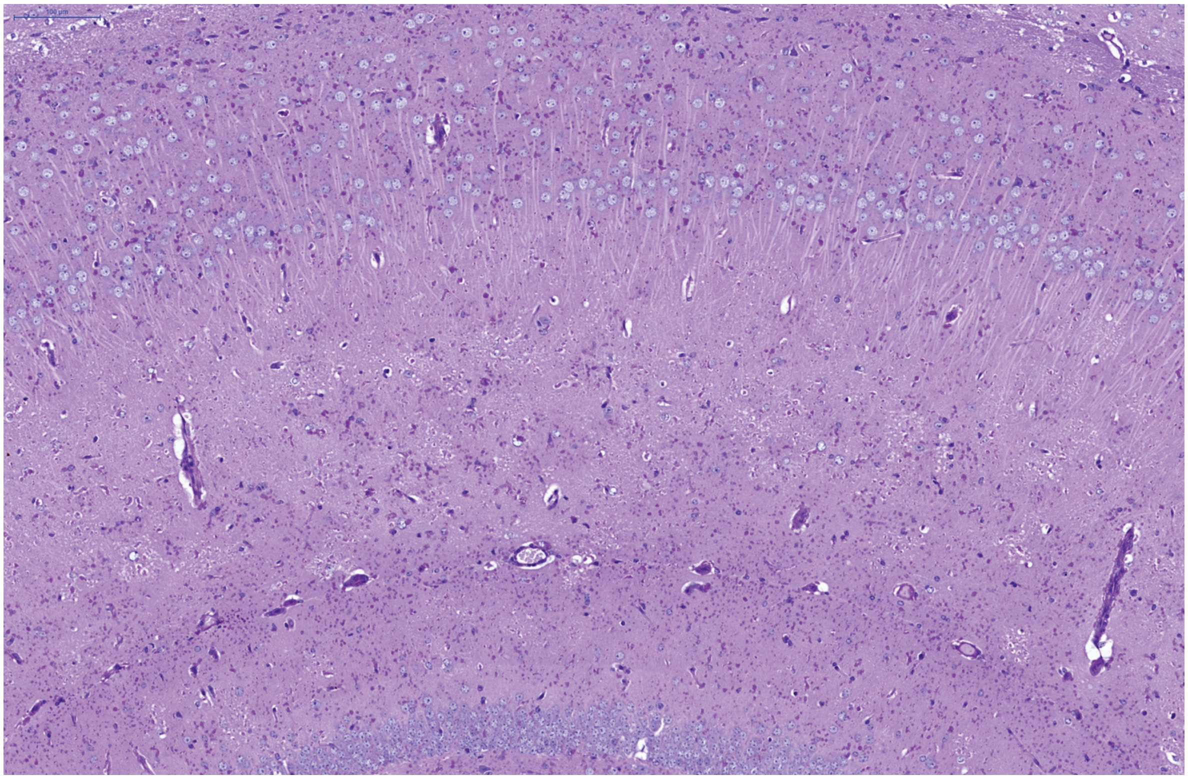

Supplement: Supplementary file 3 — Source Data for Figure 2 [file EMMM-9-906-s002.pdf]
